# Supplementary material for: Severity predictors of COVID-19 in SARS-CoV-2 variant, delta and omicron period; single center study
Source: PLoS One. 2022 Oct 25;17(10):e0273134. doi: 10.1371/journal.pone.0273134 (PMC9595523; doi:10.1371/journal.pone.0273134)
Supplement: S1 Table — (DOCX) [file pone.0273134.s001.docx]

Supporting Table 1. Patients’ Characteristics of Different Periods (Delta Period, Omicron Period) (n=141)

|  |  | Delta Period (n=89) | Omicron Period (n=52) | p value |
| --- | --- | --- | --- | --- |
| Sex (Male, %) |  | 63 (63.6%) | 36 (69.2%) | 0.851 |
| Age (y.o., Median ±SD, range) |  | 56±14.1 (18-93) | 72±15.0 (23-90) | ***<0.001 |
| Period from onset to admission (days, Median ±SD, range) |  | 7±4.1 (0-22) | 2±3.3 (0-13) | ***<0.001 |
| Period from onset to PCR positive (days, Median ±SD, range) |  | 2±1.9 (0-10) | 1±2.9 (0-13) | 0.332 |
| Symptom (cases, %) |  |  |  |  |
|  | fever | 82 (92.1) | 44 (84.6) | 0.161 |
|  | dyspnea | 56 (62.9) | 23 (44.2) | *0.032 |
|  | cough | 35 (39.3) | 19 (36.5) | 0.741 |
|  | fatigue | 27 (30.3) | 21 (40.4) | 0.221 |
|  | sore throat | 4 (4.5) | 6 (11.5) | 0.122 |
|  | consciousness disorder | 0 (0) | 1 (1.9) | 0.191 |
|  | headache | 3 (3.4) | 1 (1.9) | 0.623 |
| height (cm, median ±SD, range) |  | 167±9.4 (142-184) | 164.6±11.1 (123-189) | 0.261 |
| Weight (kg, median ±SD, range) |  | 67.4±17.7 (37.1-121) | 60.3±14.9 (34.1-107.8) | *0.027 |
| BMI (median ±SD, range) |  | 24.7±5.1 (15.7-45.5) | 23.1±4.5 (13.8-35.7) | *0.024 |
| smoking habit (cases, %) |  | 46 (51.7) | 31 (59.6) | 0.361 |
| vaccination (yes, %) |  | 6 (6.7) | 49 (94.2) | ***<0.001 |
| comorbidities (cases, %) |  |  |  |  |
|  | respiratory disease | 9 (10.1) | 11 (21.2) | 0.071 |
|  | cardiovascular disease | 7 (7.9) | 22 (42.3) | ***<0.001 |
|  | renal disease | 16 (18.0) | 24 (46.2) | ***<0.001 |
|  | continuous hemodialysis | 13 (14.6) | 17 (32.7) | *0.011 |
|  | diabetes | 20 (22.5) | 18 (34.6) | 0.122 |
|  | hypertension | 30 (33.7) | 31 (59.6) | **0.003 |
|  | hyperlipidemia | 13 (14.6) | 12 (23.1) | 0.204 |
|  | collagen diseases | 2 (2.2) | 3 (5.8) | 0.281 |
|  | with malignant tumor | 5 (5.6) | 4 (7.7) | 0.631 |
|  | pregnancy | 2 (2.2) | 0 (0) | 0.281 |
|  | immunosuppression drugs | 2 (2.2) | 5 (9.6) | 0.052 |

y.o.: year-old, SD: Standard Deviation, PCR: Polymerase Chain Reaction, BMI: Body Mass Index

Statistically significant difference **p*<0.05, ***p*<0.01, ****p*< 0.001
