# Supplementary figures and images for: Severity predictors of COVID-19 in SARS-CoV-2 variant, delta and omicron period; single center study
Source: PLoS One. 2022 Oct 25;17(10):e0273134. doi: 10.1371/journal.pone.0273134 (PMC9595523; doi:10.1371/journal.pone.0273134)

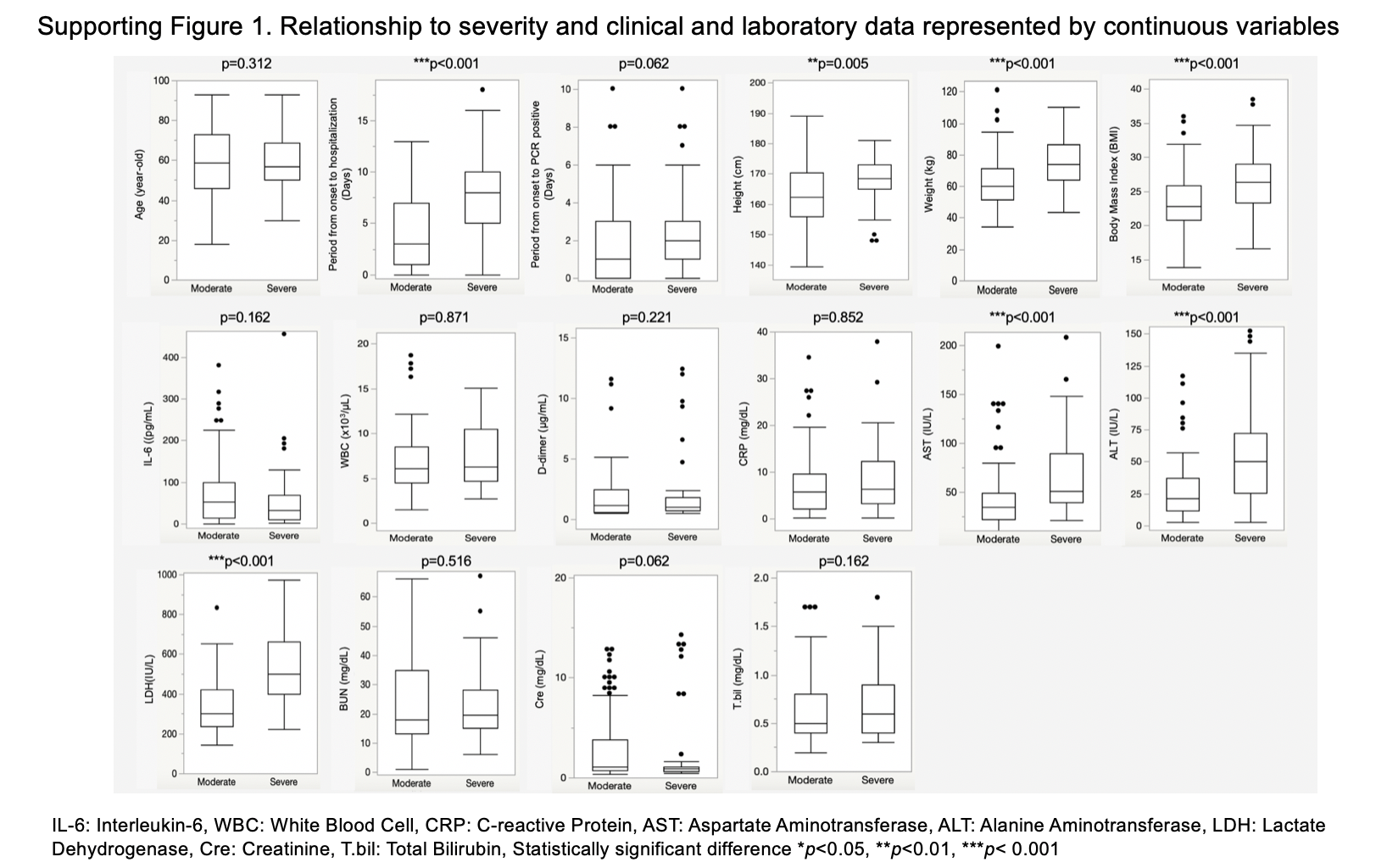

Supplement: S1 Fig — (TIF) [file pone.0273134.s003.tif]
